# The decline of measles antibody titers in previously vaccinated adults: a cross-sectional analysis

**DOI:** 10.1590/S1678-9946202466004

**Published:** 2024-01-05

**Authors:** Anna Carla Pinto Castiñeiras, Amanda Caroline Sales, Camila de Melo Picone, Constância Lima Diogo, Átila Duque Rossi, Rafael Mello Galliez, Orlando da Costa Ferreira, Terezinha Marta Pereira Pinto Castiñeiras, Marta Heloísa Lopes, Ana Marli Christovam Sartori

**Affiliations:** 1 Universidade de São Paulo, Faculdade de Medicina, Departamento de Moléstias Infecciosas e Parasitárias, São Paulo, São Paulo, Brazil; 2 Universidade de São Paulo, Faculdade de Medicina, Hospital das Clínicas, Centro de Referência para Imunobiológicos Especiais, São Paulo, São Paulo, Brazil; 3 Universidade Federal do Rio de Janeiro, Núcleo de Enfrentamento e Estudos de Doenças Infecciosas e Emergentes e Reemergentes, Rio de Janeiro, Rio de Janeiro, Brazil; 4 Universidade de São Paulo, Faculdade de Medicina, Hospital das Clínicas, Laboratório de Investigação Médica em Imunologia (LIM-48), São Paulo, São Paulo, Brazil

**Keywords:** Measles, Seroepidemiologic studies, Antibodies, Immunoglobulin G vaccination, Measles-mumps-rubella vaccine, Health personnel

## Abstract

The global reemergence of measles in 2018–2019 reinforces the relevance of high-coverage immunization to maintain the disease elimination. During an outbreak in the Sao Paulo State in 2019, several measles cases were reported in individuals who were adequately vaccinated according to the current immunization schedule recommends. This study aimed to assess measles IgG antibody seropositivity and titers in previously vaccinated adults. A cross-sectional study was conducted at CRIE-HC-FMUSP (Sao Paulo, Brazil) in 2019. It included healthy adults who had received two or more Measles-Mumps-Rubella vaccines (MMR) and excluded individuals with immunocompromising conditions. Measles IgG antibodies were measured and compared by ELISA (Euroimmun^®^) and chemiluminescence (LIASON^®^). The association of seropositivity and titers with variables of interest (age, sex, profession, previous measles, number of measles-containing vaccine doses, interval between MMR doses, and time elapsed since the last MMR dose) was analyzed. A total of 162 participants were evaluated, predominantly young (median age 30 years), women (69.8%) and healthcare professionals (61.7%). The median interval between MMR doses was 13.2 years, and the median time since the last dose was 10.4 years. The seropositivity rate was 32.7% by ELISA and 75.3% by CLIA, and a strong positive correlation was found between the tests. Multivariate analyses revealed that age and time since the last dose were independently associated with positivity. Despite being a single-center evaluation, our results suggest that measles seropositivity may be lower than expected in adequately immunized adults. Seropositivity was higher among older individuals and those with a shorter time since the last MMR vaccine dose.

## INTRODUCTION

Measles is a highly transmissible disease with thousands of cases worldwide. It can lead to serious complications, including severe diarrhea, otitis, pneumonia, encephalitis, and even death^1^. Before an effective vaccine was developed, measles epidemics occurred every two to three years, resulting in over 2.6 million annual deaths worldwide^2^. From the 1960s onwards, with the advent of the vaccine, the disease was gradually brought under control, which led to a substantial reduction in fatalities and a gradual, albeit not homogeneous, drop in cases^3^.

In the year 2000, the World Health Organization (WHO) estimated that measles caused 535,000 deaths annually and was responsible for 5.0% of all deaths of children under five, mainly in low- and middle-income countries. In 2001, a global partnership - the Measles and Rubella Initiative - was launched to ensure that no more children die of measles or are born with congenital rubella syndrome, and to assist countries in the planning, financing, and measurement of efforts required to eliminate these diseases^4^. The strategy was centered on administering two doses of measles- and rubella-containing vaccines to all children and strengthening surveillance. This collective effort resulted in the elimination of measles in the Americas, certified by the Pan American Health Organization, in 2016^5^.

The measles-mumps-rubella vaccine (MMR) is extremely effective, resulting in seroconversion in approximately 98.0% of individuals following the second dose^6^. The immune response triggered by the replication of the vaccine virus mirrors that induced by the wild-type virus, stimulating both humoral and cellular immunity, as well as interferon production. After vaccination, IgM antibodies can be detected in the organism for two to six months, while IgG persists for an extended period. The durability of the immune response following vaccination is usually influenced by the induction of cellular memory and the persistence of antibodies^7^.

Measles outbreaks in areas with high vaccine coverage tend to impact susceptible individuals. These groups include unvaccinated infants, children whose parents refuse vaccination, adults who were neither previously infected by the wild-type virus nor adequately vaccinated during childhood, and individuals with primary or secondary vaccine failure^8^.

Primary vaccine failure results from an inadequate initial response to the vaccine, which negatively affects antibody neutralization capacity and avidity^9^. Secondary vaccine failure happens due to a progressive loss of immunity over the years following vaccination, and is particularly notable in regions with low circulation of the wild-type virus^8^. This failure has been documented in cases of measles occurring in individuals with prior evidence of immunity^10-15^. Cases resulting from secondary failure tend to be milder but can still be potentially transmissible^16^.

Over the years, antibodies induced by vaccination decrease and may become undetectable^8^. This decline in antibodies does not necessarily indicate susceptibility to the virus, as an anamnestic immune response may still occur upon revaccination. However, for some individuals, this response is partial, resulting in low antibody titers, and the disease may develop. This underscores the need for surveillance during periods of viral circulation^7^.

Then, 2018 and 2019 marked a global reemergence of measles cases, even in regions that were previously free of transmission, such as Brazil. In April 2019, following virus importation from Israel and Norway into the Sao Paulo State, a new epidemic wave of measles swept the country. Sao Paulo’s metropolitan area was the epicenter, with 17,976 confirmed cases^17^. During this outbreak, infants were the most affected group in absolute numbers, accounting for 18.2% of cases and one-third of hospitalizations, while 43.1% of all cases affected individuals aged 15–29 years^18^. Notably, the disease occurred among young adults with prior vaccination, a phenomenon that had already been documented^16,19-22^.

In July 2019, the Health Department of Sao Paulo State launched a campaign to intensify measles immunization in the target population^23^. In this outbreak, as the demand for vaccination increased and an unexpectedly high proportion of cases affected vaccinated adults, we initiated a study to assess and evaluate the waning measles IgG antibodies in this vaccinated population at a vaccination reference center in Sao Paulo city.

## MATERIALS AND METHODS

A cross-sectional study was conducted from August 8^th^ to December 19^th^, 2019 at the Centro de Referencia para Imunobiologicos Especiais (CRIE) of the Hospital das Clinicas da Universidade de Sao Paulo (HC-USP). The center, established in December 1993, has served as a reference for special immunobiological and also offers routine immunization for both adults and children, following the recommendations of the National Immunization Program/Ministry of Health^24^. Its primary public includes patients, healthcare workers, and university students.

A convenience sample of participants was recruited for the study. It included individuals aged over 18 years who had visited the center for vaccine updates and had documentation proving that they had received two or more doses of the MMR vaccine, either on a physical card or electronic medical records. Those meeting these criteria were invited to participate and, upon providing written informed consent, underwent an interview and had a blood sample collected.

Data collection included information on age, gender, profession, comorbidities, medications, history of measles disease, and records of measles-containing vaccines. Each participant was assigned a protocol number to protect their personal information. Individuals with clinical conditions that could affect their immune response to vaccination, such as immunosuppression and uncontrolled diseases, were excluded.

The study primary objective was to assess the measles IgG antibody seropositivity and titers in previously vaccinated healthy adults. Additionally, the study aimed to analyze potential associations with various variables of interest (age, gender, profession, history of previous measles, number of measles-containing vaccine doses, time interval between MMR doses [in years], and time elapsed since the last MMR dose).

Measles IgG antibody titers were assessed using two widely available commercial serological tests. Initially, an enzyme-linked immunosorbent assay (ELISA) test from Euroimmun^®^ (Lübeck, Germany) was used in duplicate. Its results were interpreted according to the manufacturer’s instructions and divided into the following categories: positive (≥275 IU/L), inconclusive (≥200 to <275 IU/L), and negative (<200 IU/L). The ELISA test had a detection range of 8–5,000 IU/L and was carried out at LIM-48, a research laboratory affiliated with FMUSP.

As a counter-proof step, an automated chemiluminescent immunoassay test (CLIA) -LIAISON XL^®^ (Diasorin, Saluggia, Italy) - was subsequently performed. Results were interpreted as instructed by the manufacturer: samples were classified as positive (≥16.5 AU/mL), inconclusive (≥13.5 to <16.5 AU/mL), or negative (<13.5 AU/mL). The CLIA test detection ranged from 5 to 300 AU/mL and was conducted at the Central Laboratory of HC-FMUSP.

Data analysis was carried out using GraphPad Prism (v.9.4.1.) and RStudio (4.0.2.) softwares. The Shapiro-Wilk test was employed to assess the asymmetric distribution of variables. Continuous variables were described using the median and interquartile range (IQR), while categorical variables were presented as numbers and percentages. The Kendall Rank Correlation Coefficient test was used to evaluate the correlation between the results of the two serological tests.

For the subsequent analysis, the titers results of ELISA were considered the reference, and inconclusive samples were categorized as negative. Comparisons of antibody positivity were performed using the Mann-Whitney U test, the Fisher test, and logistic regression. IgG titers analyses were conducted using the Mann-Whitney U test, the Kendall test, and linear regression. Logistic and linear regression models were employed to assess the association of positivity and antibody titers with variables that presented a significant association in bivariate analyses. For all analyses, a p-value < 0.05 was adopted to denote statistical significance.

This research was approved by the HC-FMUSP Ethical Committee (CAPPesq Nº 3 489 630), and all participants signed a written informed consent term.

## RESULTS

From August to December 2019, 162 participants were recruited among individuals attending the CRIE-HC-FMUSP (Sao Paulo, Brazil). Table 1 presents participants’ clinical and demographic data.

**Table 1:** Demographic and clinical characteristics of 162 participants enrolled in a measles IgG antibody evaluation study. Sao Paulo, 2019.

Participants	
**Age, years**	
Median (IQR)*	30 (26-36)
(Min-Max)	(18-65)
**Gender, n (%)**	
Female	113 (69.8%)
Comorbidities, n (%) **	35 (21.6%)
**Profession, n (%)**	
Healthcare worker	100 (61.7%)
**Previous history of measles, n (%)**	13 (8.0%)
**Measles-containing vaccine doses, n (%)**	
2	60 (37.0%)
3	36 (22.2%)
4	44 (27.2%)
5	11 (6.8%)
6	9 (5.6%)
7	2 (1.2%)
**Time interval between MMR doses, years**	
Median (IQR)*	13.2 (5.6-18.5)
(Min-Max)	(0.1-26.6)
**Time since last MMR dose, years**	
Median (IQR)*	10.4 (3.6-14.1)
(Min-Max)	(0.7-27.6)

*IQR = Interquartile range (25-75%); **reported comorbidities: sickle cell anemia (1), asthma (4), depression (2), diabetes mellitus (3), celiac disease (1), endometriosis (1), centrifugal circular erythema (1), gastritis (1), systemic arterial hypertension (7), lumbar hernia (1), hyperthyroidism (1), hypothyroidism (11), rhinitis (1), rosacea (1), antiphospholipid syndrome (1), irritable bowel syndrome (1), sickle cell trait (1).

The median age of the participants was 30 years (IQR 26-36). Most were female (69.8%), White (66.8%), healthcare workers (61.7%), and declared having no comorbidities (78.4%). In total, 13 participants (8.0%) reported a history of previous measles. Most of them (86.4%) had received two to four doses of measles-containing vaccines. The median interval between MMR doses was 13.2 years (IQR 5.6–18.5), while the median time between the last MMR dose and inclusion in the study was 10.4 years (IQ 3.6–14.1).

Table 2 presents the results of ELISA and CLIA. The seropositivity rates were 32.7% by ELISA and 75.3% by CLIA. There were 20 samples (12.3%) that were inconclusive by ELISA and five (3.1%) by CLIA.

**Table 2:** Measles IgG antibody rates (%) and titers, determined by the enzyme-linked immunosorbent assay (ELISA) and chemiluminescence assay (CLIA) test in 162 adults with two or more MMR vaccine doses. Sao Paulo, 2019.

Serologic test	ELISA	CLIA
Seroprevalence, n (%)		
Positive	53 (32.7%)	122 (75.3%)
Inconclusive	20 (12.3%)	5 (3.1%)
Negative	89 (54.9%)	35 (21.6%)
IgG antibodies titers		
Median (IQR)*	182.8 (92.3-422.6) IU/L**	68.9 (17.2-190.5) AU/mL***

*IQR = Interquartile range (25-75%); **IU/L: International Units/Liter; ***AU/mL: Arbritary Units/milliliter

**Figure 1:**
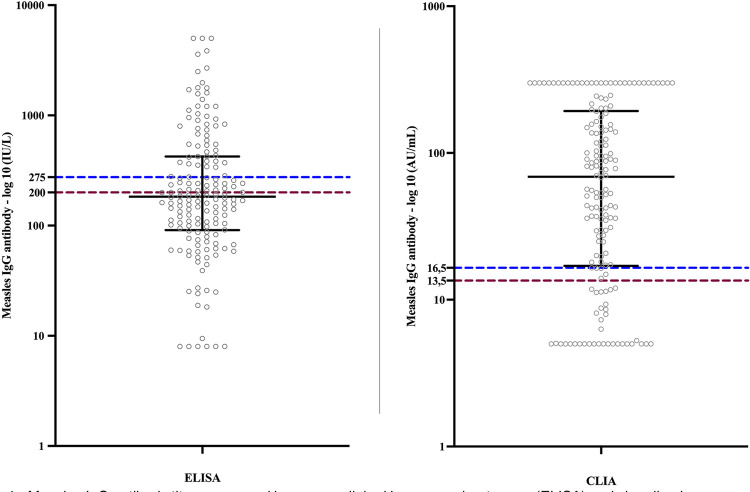
Measles IgG antibody titers measured by enzyme-linked immunosorbent assay (ELISA) and chemiluminescence assay (CLIA) tests in 162 adults with two MMR vaccine doses. Sao Paulo, 2019. Red lines represent the negative cutoff; blue lines represent the positive cutoff; black lines represent the median and IQR (25-75%).

Among the samples, 52 were positive and 34 were negative in both tests. The 20 inconclusive and 50 negative samples by ELISA were all positive by CLIA. Additionally, five samples that were negative by ELISA had an inconclusive result by CLIA, and only one sample was positive by ELISA but negative by CLIA. Figure 1 illustrates the dispersion of antibody titers in both methods. Kendall’s test revealed a strong positive correlation between ELISA and CLIA (tau coefficient 0.73; p <0.001).

**Figure 2:**
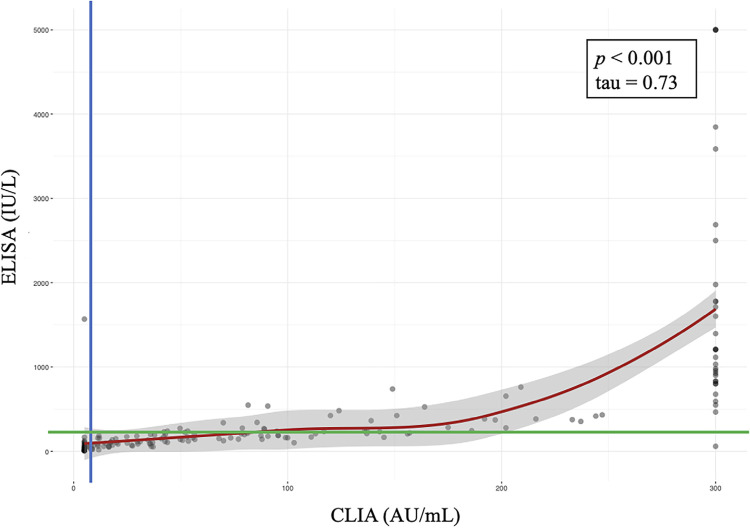
Comparison between Measles IgG antibody titers measured by enzyme-linked immunosorbent assay (ELISA) and chemiluminescence assay (CLIA) tests in 162 adults with two MMR vaccine doses. Sao Paulo, 2019. Blue lines represent the positive cutoff by ELISA; green lines represent the positive cutoff by CLIA.

Figure 2 shows the distribution of antibody titers. The overall mean measles antibody titer was 182.8 IU/L (IQR 92.3-422.6) by ELISA and 68.9 AU/mL (IQR 17.2–190.5) by CLIA. Three samples exceeded the upper limit of 5,000 IU/L in ELISA, while six negative samples had undetectable titers—and in the association analyses, the values of 5,000 IU/L and 8 IU/L were attributed to them, respectively. Thirty-one samples exceeded the upper limit of CLIA (300 AU/mL), whereas 20 had undetectable titers, and the values of 300 AU/mL and 5 AU/mL titers, respectively, were assigned to them.

Table 3 shows the associations between measles positivity rates and IgG titers with the variables of interest. In the bivariate analyses, the variables age, time elapsed since the last MMR dose, and history of measles showed a statistically significant association with both positivity and titers (p<0.05).

**Table 3:** Bivariate analyses of the association of measles IgG antibody rates and titers (ELISA) with variables of interest in 162 adults with ≥2 previous MMR vaccine doses. Sao Paulo, 2019.

	Measles IgG antibody rate (ELISA)				IgG antibodies titers	

	TOTAL	Positive	Negative ^*^	p	tau	p
Total						
n (%)	162	53 (32.7%)	109 (67.3%)	-	-	-
Age^ac^						
Median (IQR)**	29 (25-33)	33 (28-43)	29 (25-33)	**< 0.001**	0.303	**<0.001**
Measles-containing vaccine doses^ac^						
Median (IQR)**	3 (2-4)	3 (2-4)	3 (2-4)	0.4903	0.0383	0.5169
Time interval between MMR doses (years)^ac^						
Median (IQR)**	13.2 (5.6-18.5)	13.1 (7.4-16.5)	13.7 (4.4-19.3)	0.9105	0.0394	0.4566
Time after last MMR dose (years)^ac^						
Median (IQR)**	10.4 (3.6-14.1)	6.2 (2.3–1.4)	11.2 (5.6-15.0)	**0.0006**	**- 0.1998**	**0.0002**
Female^ab^						
n (%)	113	42 (37.2)	71 (628)	0.0715	-	0.1551
Healthcare worker^ab^						
n (%)	100	32 (32.0)	68 (68.0)	0.8683	-	0.3861
History of measles^ab^						
n (%)	13	9 (69.2)	4 (30.8)	**0.0102**	-	**0.0176**

^a^Mann-Whitney U test; ^b^Fisher test; ^c^Kendall test; ^*^included both negative and inconclusive samples; ^**^median [IQR: Interquartile range (25-75%)]

Logistic and linear regression models were conducted to identify factors independently associated with measles seropositivity and IgG titers, respectively (Table 4). Age was independently associated with seropositivity (OR=1.0725; 95% CI 1.024–1.1234; p=0.0031), indicating that older individuals were more likely to be seropositive for measles and to have higher antibody titers (OR=1.0216; 95% CI 1.0048–1.0629; p=0.0216). The time elapsed since the last MMR vaccine was negatively associated with seropositivity (OR=0.9421; 95% CI 0.8885–0.9989; p=0.0458), suggesting that antibody titers wane over time after vaccination. Only age was correlated with IgG titers in the multiple analyses. A history of previous measles was not associated with positivity or IgG titers.

**Table 4:** Regression analysis of the association of measles IgG antibody seropositivity and titers (ELISA) with variables of interest in 162 adults with two MMR vaccine doses. Sao Paulo, 2019.

	Measles IgG antibody seropositivity (ELISA)*			IgG antibodies titers**		
	
	OR	95% CI	p	Regression coefficients	Standard error	p
Age	1.0725	1.024-1.1234	**0.0031**	25.8	7.9	**0.0014**
Time after last MMR dose	0.9421	0.8885–0.9989	**0.0458**	-8.8	9.9	0.3747
Previous history of measles	3.2019	0.8597-11.9250	0.0828	367.3	245.0	0.1359

^*^logistic regression; ^**^linear regression

## DISCUSSION

This study was conducted during a measles outbreak in the Sao Paulo State in 2019 and aimed to assess measles IgG antibody titers among adults who had previously received at least two MMR vaccine doses after one year of age. Two widely distributed commercial serological tests, ELISA and CLIA, were employed. ELISA identified 32.7% of the samples as positive (≥275 IU/L), 12.3% as inconclusive (≥200 to <275 IU/L), and 54.9% as negative (<200 IU/L). On the other hand, CLIA indicated that 75.3% of the samples were positive (≥16.5 AU/mL), 3.1% were inconclusive (≥13.5 to <16.5 AU/mL), and 21.6% were negative (<13.5 AU/mL). Notably, these two tests showed a strong positive correlation with a tau coefficient=0.73 (p<0.001).

According to the manufacturer’s instructions, the CLIA positive cutoff (16.5AU/mL) is equivalent to 175 IU/L (as per the WHO Third International Standard for Anti-Measles). This suggests that the CLIA seropositivity cutoff is lower than that of ELISA (≥275 IU/L), which partly explains the higher proportion of seropositivity indicated by CLIA. This hypothesis is further supported by the fact that all samples classified as inconclusive by ELISA tested positive by CLIA.

A Thai study^25^ compared ELISA antibody titers with protective neutralizing antibodies (>120 mIU/mL) in children and adolescents aged 3 to 18 years. They found a 100% correlation when the ELISA cutoff was set at ≥275 IU/L, an 85.7% correlation when it was set at ≥200 IU/L, and a 72.2% correlation when it was set at >120 IU/L. When using the cutoff recommended by Euroimmun^®^ (≥275 IU/L), they found a positivity rate of 46.3%, which is more similar to our findings. In the Thay study, an inverse correlation was observed between antibody titers and age, with protection declining with aging. This trend was partially attributed to the collinear relationship between increasing age and the absence of vaccine records among participants.

Another study compared CLIA (LIAISON XL^®^) with PRNT^26^. CLIA exhibited a 90.2% sensitivity rate (95% CI 82.7–79.2) and a 75.0% specificity (95% CI 59.7-86.8). The comparison revealed a disagreement of 14.4% between the tests, which was more frequent near the lower cutoff. The authors noted that CLIA may yield false-negative results in the vaccinated population, which leads to an underestimation of protection against measles.

An American study aimed to validate commercial immunoassays and employed ELISA (Euroimmun^®^) and CLIA (LIAISON XL^®^) to evaluate measles antibodies titers compared to neutralization tests^27^. Both tests showed a positive correlation with neutralization, but ELISA demonstrated a stronger correlation (R=0.71–0.79; p<0.0001) than CLIA (R=0.40–0.55; p<0.05) and yielded more precise results.

Antibody titers tend to decline over the years after vaccination. In 2011, a study evaluated 764 adolescents and young adults aged 11–22 years who had received two doses of the MMR vaccine using an automated plaque reduction microneutralization (PRMN) assay. The study revealed that 8.9% of participants had non-protective neutralizing antibody titers (titers <120 mIU/mL). This percentage was interpreted as indicating potential susceptibility to symptomatic disease^28^. Furthermore, a 2020 meta-analysis estimated an annual antibody decline rate of 0.009 in a similar population, implying that 8.6% of initially positive individuals would transition to a negative status over 10 years^8^. Another study from the US revealed an even more pronounced decrease in neutralizing antibodies, projecting a 33.0% seronegative rate after 20 years of MMR immunization^29^.

As vaccination coverage expands and the number of unvaccinated individuals decreases, the proportion of vaccinated individuals among those with confirmed measles cases is expected to rise^30^. In fact, approximately 40.0% of people infected with measles in the 2019 Sao Paulo outbreak had a history of previous vaccination^20^. Those aged 20 to 35 years were the most affected, accounting for 40.2% of cases^31^. This age group, born after the implementation of systematic measles vaccination, observed multiple changes in the vaccination schedule and comprised the majority of participants in our study (median 30; IIQ 26–36 years).

In our study, the variable age was independent and positively associated with seropositivity (p=0.0024) and higher IgG titers (p=0,0014). This result can be partly attributed to underreported infections and higher immunogenic stimulus due to repeated wild virus exposure in older individuals. A meta-analysis examining the effects of age and gender on measles susceptibility found that individuals born before 1980 had a 2.78 relative risk (RR) (95% CI 2.18–3.50; p<0.0001) of being seropositive compared to younger individuals. No significant association with gender was found (RR=0.92, 95% CI 0.83–1.03, p=0.02)^32^.

Healthcare professionals did not show a significant association with seropositivity (p=0.8683). Note that, in addition to having an individual risk of infection, these professionals can become a source of nosocomial infection themselves, potentially exposing a population that lacks immunity, making them vulnerable to severe disease. This may lead to increased measles morbidity and mortality^33-35^.

In South Korea, a study conducted during an outbreak in 2007 found that nearly half of cases occurred in a hospital environment^34^. The authors also noted that nosocomial spread preceded the peak of the community outbreak by approximately two weeks. Notably, 23.0% of the healthcare professionals affected during the South Korea outbreak had previously received two MMR doses, which emphasizes the need to assess their measles immunological status.

Surprisingly, in our study, prior measles infection was not independently associated with seropositivity (p=0.0828) or antibody titers (p=0.1359) by the ELISA method. However, only one participant out of the four who had a history of measles and a negative antibody titer in ELISA was also negative in CLIA. It is also noteworthy that since measles mainly affects children, recall bias may affect the reliability of information on cases, and measles may be misdiagnosed with other childhood exanthematous diseases. Similarly, the number of measles vaccine doses was subject to measurement bias. While some participants had proof of vaccination since childhood, others only had adult life records.

The interval between MMR doses was relatively long (median 13.2, IQR 5.6–18.5 years) and did not differ significantly between seropositive and seronegative individuals (p=0.9105). This extended interval may be related to changes in vaccination schedules over time and is expected to decrease in the coming years, due to the systematic recommendation of two MMR doses in childhood.

In contrast, the time elapsed since the last MMR dose did emerge as a negative independent predictor for seropositivity (OR=0.9421, 95% CI 0.8885–0.9989, p=0.0458). The median time since vaccination was greater in seronegative individuals than in seropositive ones: 11.2 (IQR 5.62–15.0) and 6.15 years (IQR 2.29–11.4) (p=0.0006), respectively. A previous German study^36^ also observed this association, indicating that individuals with more than eight years elapsed since the last dose were 4.59 times more likely to be seronegative than those vaccinated within the last two years.

The high rate of seronegative individuals in adequately vaccinated populations raises concerns about the potential role of a third dose of the MMR vaccine, particularly during outbreaks. An American study^37^ observed a significant increase in neutralizing antibodies after a booster dose in young adults. Most individuals with non-protective baseline titers (<120 mIU/mL) seroconverted after a third dose, but returned to near-baseline titers after one year.

The booster effect may help disrupt the transmission chain and achieve disease control, albeit temporarily. Therefore, while periodic boosters of measles-containing vaccines may not be justified, they may be valuable during outbreaks.

This study aimed to expand the knowledge about measles susceptibility in Sao Paulo City, potentially guiding measures to control outbreaks and effectively eliminate the disease. However, it is essential to acknowledge certain limitations that may have partially compromised the accuracy of the results. The study population was selected by convenience sampling, and the sample size may be insufficient to identify some associations.

The reported cases of measles were not laboratory-confirmed, and precise dates of illness onset were undocumented. However, none of the patients had contracted the illness during the most recent outbreak. Antibody measurements were conducted using immunoassay tests rather than the gold standard (PRNT). In addition, the laboratory variation in test procedures can make it challenging to compare the results.

Data on measles seroprevalence during periods of viral circulation may be relevant for estimating the impact of the outbreak^7,29^. Our data revealed that 67.3% of young adults considered adequately vaccinated had apparently non-protective IgG antibody titers, which may render them susceptible to measles. Nonetheless, it is important to emphasize that a low number of IgG titers or even the absence of them do not necessarily imply a lack of protection upon virus exposure. Vaccines can also stimulate cellular immune responses similar to, albeit less pronounced than, the wild-type virus^7^. Although not currently detectable with available tests, this response may still provide protection against the disease.

Future studies could expand the knowledge on measles protection in the general population and specific groups. Employing the gold standard measles neutralization test would validate the results obtained with immunoassay tests. The long-term persistence of antibodies should be evaluated, especially in cohorts exclusively vaccinated during early childhood. Furthermore, the immunological, epidemiological, and financial aspects of a potential third measles-containing vaccine dose need to be studied, particularly during outbreaks.

Lastly, the elimination of regional measles must be seen as a fundamental step toward global eradication. However, it is crucial to recognize its fragility. Sustaining elimination requires maintaining high and homogeneous vaccination coverage and improving surveillance to prevent virus importation and a subsequent measles spread^38^.

## CONCLUSION

This study found that 67.3% of young adults previously vaccinated with two or more MMR vaccine doses were seronegative by ELISA after a median of 10.4 years. These findings suggest that current measles susceptibility, in times when immunity depends essentially on vaccine stimuli, could be higher than expected and should be better elucidated by further studies. The main factors associated with waning immunity titers were age and time elapsed since the last MMR dose.

In light of the increasing global incidence of measles, our results highlight the importance of periodically reassessing vaccination strategies and recommendations, particularly during periods of heightened measles transmission.
